# A Novel Deep Learning Method for Recognition and Classification of Brain Tumors from MRI Images

**DOI:** 10.3390/diagnostics11050744

**Published:** 2021-04-21

**Authors:** Momina Masood, Tahira Nazir, Marriam Nawaz, Awais Mehmood, Junaid Rashid, Hyuk-Yoon Kwon, Toqeer Mahmood, Amir Hussain

**Affiliations:** 1 Department of Computer Science, University of Engineering and Technology, Taxila 47050, Pakistan; momina.masood@uettaxila.edu.pk (M.M.); tahira.nazir77@gmail.com (T.N.); marriam.nawaz@uettaxila.edu.pk (M.N.); awais.mehmood@uettaxila.edu.pk (A.M.); 2 Department of Computer Science, AIR University Islamabad, Aerospace and Aviation Campus Kamra, Kamra 43570, Pakistan; 3 Department of Industrial Engineering, Seoul National University of Science and Technology, Seoul 01811, Korea; 4 Department of Computer Science, National Textile University, Faisalabad 37610, Pakistan; toqeer.mahmood@yahoo.com; 5 Data Science and Cyber Analytics Research Group, Edinburgh Napier University, Edinburgh EH11 4DY, UK; a.hussain@napier.ac.uk

**Keywords:** MRI, brain tumor, Mask-RCNN, deep learning

## Abstract

A brain tumor is an abnormal growth in brain cells that causes damage to various blood vessels and nerves in the human body. An earlier and accurate diagnosis of the brain tumor is of foremost important to avoid future complications. Precise segmentation of brain tumors provides a basis for surgical planning and treatment to doctors. Manual detection using MRI images is computationally complex in cases where the survival of the patient is dependent on timely treatment, and the performance relies on domain expertise. Therefore, computerized detection of tumors is still a challenging task due to significant variations in their location and structure, i.e., irregular shapes and ambiguous boundaries. In this study, we propose a custom Mask Region-based Convolution neural network (Mask RCNN) with a densenet-41 backbone architecture that is trained via transfer learning for precise classification and segmentation of brain tumors. Our method is evaluated on two different benchmark datasets using various quantitative measures. Comparative results show that the custom Mask-RCNN can more precisely detect tumor locations using bounding boxes and return segmentation masks to provide exact tumor regions. Our proposed model achieved an accuracy of 96.3% and 98.34% for segmentation and classification respectively, demonstrating enhanced robustness compared to state-of-the-art approaches.

## 1. Introduction

A brain tumor is a fatal disease-causing death to thousands of people around the globe. A brain tumor is mainly caused by abnormal growth in brain tissues. As the skull portion of the human body is inflexible and small, any growth inside the brain may affect the functionality of the human organ depending on its origin and position. Moreover, it may also spread in other parts of the body and affect their functionality [1]. Usually, the brain tumor is categorized into two classes, named primary and secondary based on its position. The primary tumor comprises 70% of all brain tumors while the remaining 30% are secondary [2]. A primary brain tumor includes tumors that originate from the brain cells while a secondary brain tumor first originates in another organ and then transfers to the brain through the circulation of the blood [3]. According to an NBTF study, in the USA an estimated 29,000 cases are diagnosed with primary brain tumor, among which, around 13,000 patients die per year [4]. Similarly, in the UK, more than 42,000 patients with a primary brain tumor die annually.

Among various primary brain tumor types, glioma has the highest mortality and mobility rate [5]. Gliomas usually grow from glial cells of the brain [1] and are classified as low-grade (LG) glioma and high-grade (HG) glioma. The HG glioma is more life-threatening and intense and usually, the victim can survive for two years [6]. A meningioma tumor usually develops in the protective membrane layer which acts as a covering of the human brain and spinal cord. Mostly, meningioma tumors are less threatening and slow-growing [7]. The pituitary tumor starts developing in the pituitary gland, which is located at the base of the brain and is involved in the production of several essential hormones in the body [8]. A pituitary tumor is a benign tumor; however, serious complications may cause hormonal deficiencies or permanent loss of vision because of the overproduction of hormones [1]. Hence, an early-stage detection of brain tumors is critical and is of extreme clinical interest. If it is not diagnosed on time, the disease could become life-threatening or may result in the disability of a person [7].

Depending on the situation and their purpose, numerous medical imaging techniques can be used in clinical practices for tumor diagnosis [9]. Some of those techniques are ultrasonography (US), magnetic resonance imaging (MRI), and computed tomography (CT) [10]. The MRI is the most common non-invasive imaging technique because it uses no damaging ionizing radiations of X-rays during the scan. Moreover, it provides high-quality images of soft tissue without any risk, plus an ability to acquire multiple modalities, e.g., T1, T1c, T2, and FLAIR, using various parameters. Each of these modalities produces noticeably a unique tissue contrast [11].

For timely treatment, the top priority of a neurosurgeon is to mark out tumor regions as precisely as possible, otherwise excessive or insufficient cutting may lead to suffering or a permanent loss. Unfortunately, this manual segmentation process is laborious and time-consuming and yields poor segmentation results [12]. Hence, the use of computer-aided brain tumor segmentation algorithms using MRI to identify and segment brain tumors has received considerable attention from the research community. Therefore, there is a significant need for automated and efficient tumor detection and segmentation technique. Despite recent developments in automatic or semi-automatic techniques for tumor segmentation, it is still a challenging task to segment a tumor accurately because of the following reasons [13]. First, there is a significant change in tumor location, shape, appearance, and size from patient to patient [14]. Second, the tumor boundaries can be discontinuous or blurry as the tumor regions are usually occupied by surrounding healthy tissues [15]. Third, the addition of inadequate signal-to-noise ratio or image distortion usually caused by different factors such as MRI acquisition protocols or variation in imaging devices may further increase the difficulty and influence the precision of final segmentation [16].

The brain tumor detection approaches can be divided into two types, named machine learning (ML)-based [17] and deep learning (DL)-based [18] methods. ML-based techniques mainly include support vector machine [19], conditional random forest [20], decision tree [21], principal component analysis [22], and fuzzy c-means [23]. These techniques require hand-crafted features. The hand-crafted features, here, mean that the features are required to be extracted from training images to start the learning process and perhaps require an expert with extensive knowledge to identify the most important features. Hence, the detection accuracy of the ML-based techniques is dependent on the quality and representation of the extracted features, thus is limited and prone to errors in dealing with large datasets [24]. Meanwhile, DL-based algorithms have shown high performance in various industries including medical imaging [25,26,27]. The most common or well-known DL model is the convolutional neural network (CNN) that can instinctively learn dense characteristics directly from the training data due to its weight-sharing nature [28]. Based on these advantages, DL-based brain tumor segmentation has grabbed the researcher’s attention [29]. Relevant works include patch-based CNN [30], patch-based multi-scale CNN [31], patch-based DCNN [32], fully convolution-based CNN (FCNN) [33], and U-net based [34] brain tumor segmentation models. The patch-based approaches take a small portion of the image as input to CNN and classify each patch into a different class, which degrades the image content and label correlation. The FCNN, on the other hand, is a modified form of CNN, which predicts probability distribution pixel-wise instead of making patch-wise probability distribution predictions [35]. This improvement enables FCNN to take the full-sized image and perform the prediction for the whole image in just a single forward pass. Despite recent advances, the existing DL-based techniques require several convolution layers (CLs) and kernels, increasing the computational cost resultantly. Hence, an efficient method for accurate tumor identification and segmentation with a less complex network in terms of memory and computing resources usage is still in demand [36].

In this paper, we proposed an automated approach for brain tumor detection and segmentation using MRI images. The proposed technique adopts a DL model using a fully convolution neural network, Mask-RCNN [37] with DenseNet-41 backbone, and utilizes a multitask loss function to achieve an end-to-end training of deep CNN, increasing the detection accuracy. The motivation behind using a custom Mask-RCNN was to achieve a similar level of accuracy with a comparatively simple model, fewer kernels, and two convolutional layers. To show the efficiency of the proposed approach, we evaluated our model on free and online available brain tumor datasets [38,39] using various quantitative measures. The results of brain tumor MRI segmentation are validated through ground truth analysis. The recent works have investigated the effectiveness of a Mask-RCNN model on 3D images of the brain [40] and in other medical fields such as oral disease [41], breast tumor [42], and lung tumor [43] detection and segmentation. The main contributions of the proposed work are as follows:

1. The proposed method can precisely segment and classify the brain tumors from MRI images under the presence of blurring, noise, and bias field-effect variations in input images.
2. We have created the annotations which are essential for the training of the proposed model because available datasets do not have a bounding box and mask ground truths (GTs).
3. The accurate localization and segmentation of tumor regions due to an effective region proposal network of DenseNet-41-based Mask-RCNN as it works in an end-to-end manner.
4. Extensive experiments are performed using two different datasets to show the robustness of the presented framework and compared obtained results with the existing state-of-the-art methods.

The rest of our paper develops the following structure: the literature review is explained in Section 2, while the brief description of the proposed work is defined in Section 3. In Section 4, the datasets, evaluation parameters used, and experimental results obtained are reported. Finally, a conclusion of this work is described in Section 5.

## 2. Related Work

Due to the high clinical importance and the complex nature of the brain tumor, the advancement of an automatic model is an active research area. This section briefly discusses the relevant works for brain tumor classification and segmentation from MR images. Initially, the ML approaches such as support vector machines vector machines [19], conditional random forests [20], decision forests [21], principal component analysis [22], and fuzzy c-means [23] were presented. A common aspect of these methods is that they classify image voxels based on the pre-defined feature set known as handcrafted features, which require a human expert to figure out the most promising features from training images to start the training process.

In recent years, DL-based approaches have exhibited encouraging results in the automatic segmentation of medical imaging [25]. The most important aspect of DL approaches is that they can learn complex feature representation automatically from the training data and thus result in a more robust feature vector. Various DL models for the automatic recognition of tumors have been presented, achieving promising results [30,31,32,33,34,36,44]. Pereira et al. [30] trained two different 2D CNN with deeper layers as a sliding window classifier for the segmentation of both LG and HG glioblastomas. Urban et al. [45] proposed a two pathway-based 2D CNN approach on large patches to incorporate both global and local information. A local path focuses on the information in neighbor pixels while a global path captures larger contextual information simultaneously from MRI. Kamnitsas et al. [32] introduced a 3D CNN architecture that considers 3D patches along with global contextual features through down-sampling, as post-processing, a fully connected CRF network was employed. In [46], the extended form of DeepMedic by adding residual connection is proposed for tumor segmentation. These above-mentioned approaches built using CNN for brain tumor segmentation operate at patch-level and consider local regions in MRI images which are then used to classify each patch [47]. Based on the obtained classification results, the central pixel is labeled; thus, it only explores spatially limited contextual information.

Recently, FCNNs have achieved promising results for the segmentation of natural images [35] as well as medical images [36]. In FCNN, convolutional kernels are employed instead of fully connected layers. The original size of the image is restored by using up-sampling and deconvolution layers. Moreover, the model is trained end-to-end and has computational efficiency over patch-level classification approaches. Havaei et al. [31] proposed an architecture, named InputCascadeCNN, which passes pixel-wise probability estimates obtained by first CNN as an additional input to the following one. This two-stage training strategy solves an imbalance of label distribution and captures multi-scale features using a multi-cascaded network. Zhao et al. [33] presented a unified framework by integrating FCNNs with CRFs, such that the results reported after the segmentation of tumors maintain their appearance and spatial consistency. In [28], a multi-cascade convolutional neural network was proposed that takes both local pixel dependencies and more discriminative multi-scale features of 3D MRI images into account. To further refine the obtained results, CRFs are used to smoothen the tumor edges and eliminate false positives. In [36], authors developed the U-Net an encoder–decoder-based mod which consists of a regular FCNN followed by a contracting path capturing contextual features by down-sampling at each layer and an expanding path which raises the image size by up-sampling at each layer, thus enabling precise localization and segmentation, and it is well suited for the segmentation of medical imaging [48]. Don et al. [34] adopted a U-net CNN architecture for the segmentation of a tumor with some minor modifications. They used the technique of data augmentation with dice-based loss function to improve segmentation accuracy.

In [49], a watershed segmentation algorithm was used to segment a brain tumor. However, they employed low dimensional hand-crafted feature vectors as training to the KNN classifier for classification. They achieved an average accuracy of 86%. In [50], an encoder–decoder-based architecture is presented, which performed the pixel-wise segmentation of tumor tissues from normal brain cells. This model used SegNet architecture with an encoder of depth four and VGG16 for generating feature maps, and performed non-linear up-sampling. This approach does not need any post-processing phase and reported an average dice score of 0.931. In FR-MRINet [51], a 33-layer deep model and an encoder with a fully connected decoder instead of the deconvolutional decoder were proposed. However, the approach produces anomaly areas, i.e., small non-tumor areas are predicted to contain tumors and cleaned by using the neighborhood cleaning rule. This work reported a segmentation accuracy of 91.4%.

In this work, we adopt a kind of DL method, Mask-RCNN [37] architecture, that uses end-to-end training for the localization and classification of brain tumors. Instead of applying a threshold-based or boundary-based model for accurate segmentation, this method uses region-based segmentation and generates a mask that achieves improved tumor boundary segmentation accuracy.

## 3. Proposed Methodology

**Figure 1:**
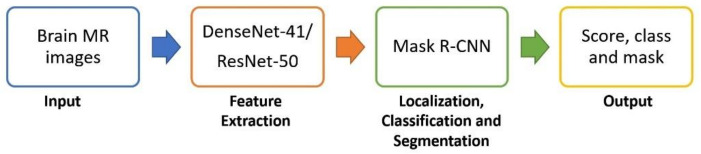
Block diagram of the proposed method.

This section illustrates the architecture of our presented method employed for brain tumor detection. Figure 1 shows the workflow of the proposed methodology. The presented DL technique is DenseNet-41-based Mask-RCNN, which aims to perform the accurate localization, segmentation, and classification of brain tumors. Given an MRI image, our aim is to instinctively detect the brain tumor from a complex background without requiring any manual intrusion. First, the input images are preprocessed to remove noise and artifacts added during MRI acquisition. Then, the ground truth segmentation masks are generated that are utilized for model training. Next, custom Mask-RCNN is applied for tumor localization, classification, and segmentation. The custom Mask-RCNN model performs the following steps: (1) keypoints extraction using DenseNet-41, (2) region of interest (RoI) creation, (3) RoI classification and bounding box regression, and (4) segmentation mask acquisition. First, a backbone network based on CLs computes the deep features from preprocessed input MRI images. The obtained features are then used by the region proposal network (RPN)to generate RoIs by mapping each point on the feature map into the original image. Next, the RoIAlign layer is employed to select features from the feature map corresponding to the RoIs obtained from the RPN network and distribute them with correlated layers to segment and classify the RoI.

### 3.1. Preprocessing

The MRI images produced from different MRI machines could have a bias field or intensity inhomogeneity, which is an artifact and should be corrected as it affects the segmentation results [52]. In the preprocessing step, we applied the level set method for bias field correction [53]. To obtain the enhanced image, a median filter was applied. The median filter is considered better as compared to linear filtering for eradicating noise [54].

### 3.2. Annotations

**Figure 2:**
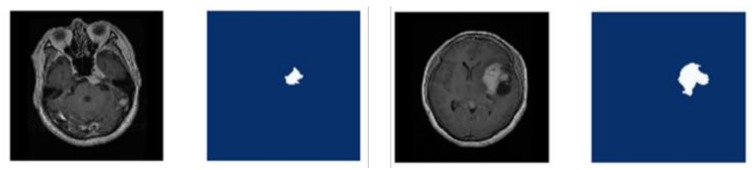
Sample original images and corresponding ground truth masks.

The GT mask associated with every MRI image is essential to distinguish the tumor portion for the training procedure. The VGG Image Annotator (VIA) [55] is utilized to annotate the MRI images and then produce a polygon mask for every image. Figure 2 indicates an instance of the original image and the related GT image. The VIA interpretations are saved in a JSON file which comprises the set of polygon points for the tumor region and a value of region attributed 0 or 1. The pixels inside the bounding polygon are related to a tumor region and are given a value of 1 while the rest of the pixels are regarded as background and assigned the value of 0. This file is used to create a mask image corresponding to each MRI image that is later used in the training process.

### 3.3. Tumor Localization and Segmentation Using Mask-RCNN

**Figure 3:**
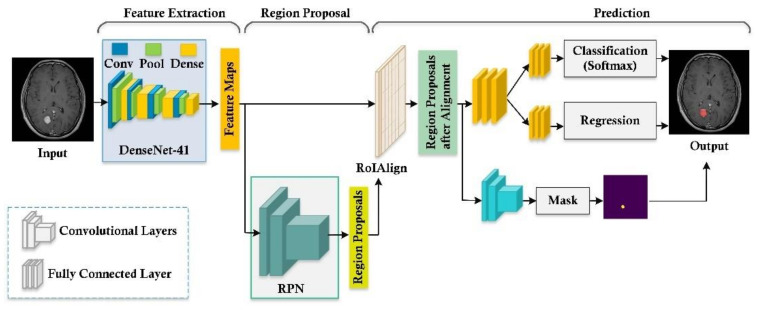
The structure of the proposed technique.

Mask-RCNN is the recent DL technique that is used for both object detection and pixel-level segmentation [37]. It is the extension of Faster RCNN [56], which performs segmentation as well along with classification and localization. Furthermore, Mask-RCNN decouples the mask and class prediction. It adds a small network overhead, i.e., an FCN network to perform segmentation. In the presented work, we proposed a custom Mask-RCNN by introducing the DenseNet-41 at the feature computation layer. DenseNet [57,58] is the latest presented approach of CNN, in which the present layer relates to all preceding layers. DenseNet comprises a set of dense blocks that are sequentially interlinked with each other with extra convolutional and pooling layers among successive dense blocks. DenseNet can present the complex transformations which result in improving the issue of the absence of the target’s position information for the top-level key points to some degree. DenseNet minimizes the number of parameters, which makes them cost-efficient. Moreover, DenseNet assists the keypoints propagation process and encourages their reuse, which makes them more suitable for brain tumor classification. The structure or flow of the presented approach is illustrated in Figure 3. The custom Mask-RCNN architecture consists of different networks, i.e., a convolutional backbone network, region proposal network, RoI classifier and bounding box regressor, and the segmentation network. A detailed description of each step is discussed below.

#### 3.3.1. Feature Extraction

The backbone network is employed to obtain the relevant feature from the input MR images [59]. This network could be any CNN model intended for image analysis, such as ResNet-50, ResNet-101, and DenseNet. A keypoints extraction network must be intensely adequate with many convolution layers such that it can appropriately learn reliable and more discriminating features. However, the increase in network depth size adds more computational overhead and makes it difficult to optimize the network weights, which may result in an exploding gradients problem. The work in history has employed ResNet for medical image analysis with Mask-RCNN. However, the ResNet model uses skip-connections and comprises many parameters, which eventually results in the vanishing gradient problem. In the presented work, we have implemented Mask-RCNN with both the ResNet and DenseNet framework. More specifically, we have computed features with ResNet-50 and DenseNet-41 frameworks. Since DenseNet contains dense connections, this results in computing a more representative set of image features.

**Figure 4:**
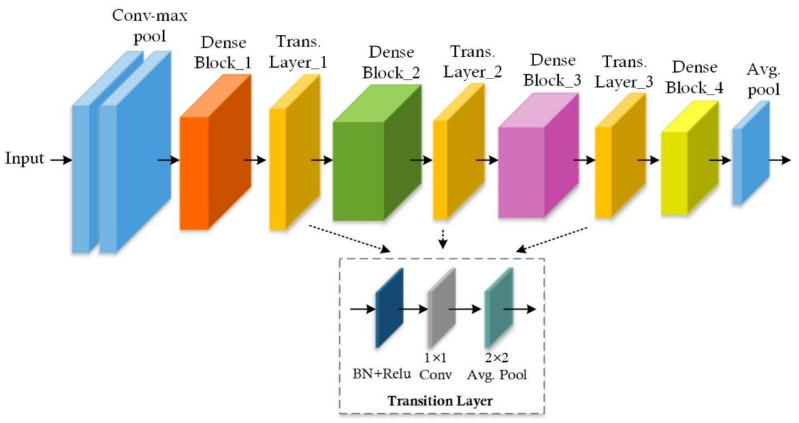
DenseNet-41 architecture.

The DenseNet-41 has two potential differences from the traditional DenseNet: (i) DenseNet-41 has fewer parameters from the actual model as instead of 64, it has 24 channels on the first convolution layer and the size of the kernel is 3 × 3 instead of 7 × 7; (ii) the number of layers within each dense block is attuned to deal with the computational complexity. The dense block is the fundamental part of DenseNet-41, as shown in Figure 4.

#### 3.3.2. Region Proposal Network

In this stage, the feature map obtained by feature extraction is fed to the RPN network to generate RoIs. The RoIs are localized to tumor regions used for the final segmentation and classification. The RPN module uses a 3 × 3 CL to scan the whole image in a sliding window manner to generate relevant anchors. These anchors are the bounding box with different sizes and are distributed over the whole image. Since there are about 20 k anchors of various sizes and scales, they are likely to overlap each other to cover the whole image as much as possible [60]. Using RPN prediction, the top anchors that probably include objects are selected and their position and size are refined using bounding box regression. In the case of the overlapping anchors, the ones with the highest foreground score are kept while the remaining ones are discarded by using non-max suppression. More specifically, if an anchor has an intersection-over-union (IoU) higher than 0.7 with a GT box, it is classified as a positive anchor (fg class), otherwise negative (bg class). This leads to a generation of several RoIs that are passed to the next stage for classification and segmentation.

#### 3.3.3. RoI Classification and Bounding Box Regression

This network takes the proposed RoI and feature map as input (Figure 3). Unlike the RPN, which returns two classes such as foreground and background, this network is deeper and classifies proposed RoIs to a specific class, i.e., glioma, meningioma, and pituitary, and further improves the size of the bounding box. This step intends to pool all RoIs on the feature maps to a fixed size. Usually, the boundaries of RoI do not coincide with the granularity of the feature map as the feature map is down-sampled k times from the size of the original image (via convolutions). To resize the feature maps, the RoIAlign layer is utilized to obtain the fixed length of keypoint vectors for arbitrary-size candidate regions and performs the bilinear interpolation to avoid misalignment issues encountered in the RoI pooling layer, which uses quantization operation. These keypoints are fed into categorical classification and regression layers to get the ultimate recognition results.

#### 3.3.4. Segmentation Mask Acquisition

In this step, the mask branch works separately from the classification and regression. The segmentation network takes positive regions chosen by the RoI classifier as input and returns a segmentation mask of 28 × 28 resolution. The obtained masks are represented by floating numbers and thus contain more information as compared to binary masks. The GT masks are scaled down to a size of 28 × 28 to measure the loss with the predicted mask during the training stage. However, during the inference phase, the predicted mask is scaled up to match the dimensions of the RoI bounding box and which provides the final output mask.

### 3.4. Loss Function

During training, the Mask-RCNN model [37] employs a multi-task loss L on each sampled RoI, defined as:

(1) $$L\left(Mask\;RCNN\right)=L_{class}+L_{bbox}+L_{mask}$$

where *L_class_*, *L_bbox_*, and *L_mask_* represent the class label prediction loss, bounding box refinement loss, and segmentation mask prediction loss, respectively.

$L_{class}$ is defined as follows:

(2) $$L_{class}=-\log P_{u}$$

where *P* is a (k + 1) dimensional vector corresponding to the likelihood of a pixel going to the k class or background. For each RoI, $P=P_{0},\ldots ,P_{k}$ and $P_{u}$ is the probability related to class *u*.

$L_{bbox}$ is defined as follows:

(3) $$L_{bbox}\left(v_{i},\;{v_{i}}^{*}\right)=\underset{i\in \left\{x,\;y,w,h\right\}6}{\sum }smooth_{L1}\left(v_{i}-v_{i}^{*}\right)$$

where

(4) $$smooth_{L1}\left(x\right)=\left\{\begin{array}{cc} 0.5x^{2} & \mathrm{if}\;\left|x\right|<1\\ \left|x\right|-0.5 & \mathrm{otherwise}, \end{array}\right.$$

Vector vi represents four parameters coordinate of the predicted bounding box, and ${v_{i}}^{*}$ is the coordinate of the GT relating to the positive anchor. The smooth-*L*_1_ function is a robust *L*_1_ loss that is less sensitive to outliers over *L*_2_ loss. When regression targets are infinite, training with *L*_2_ loss can necessitate the careful modification of learning levels to avoid exploding gradients. For the training of the mask network, the average binary cross-entropy loss is employed that is given as follows:

(5) $$L_{mask}=-\frac{1}{n^{2}}\underset{1\leq i,\;j\leq n}{\sum }\left[x_{ij}\cdot logP_{ij}^{k}+\left(1-x_{ij}\right)\cdot log\left(1-P_{ij}^{k}\right)\right]$$

where $x_{ij}$ represents the value of a pixel $\left(i,j\right)$ in a GT mask of size n×n and $P_{ij}^{k}$ is the predicted value of the same pixel in the mask learned for class *k*.

## 4. Performance Evaluation

### 4.1. Experimental Setup

For implementation, we used the Mask-RCNN model provided by Matterport Inc. [60] released under MIT license based on open-source Tensorflow and Keras Libraries. The Mask-RCNN model with both ResNet-50 and DenseNet-41 frameworks was implemented. In our work, rather than training our model from scratch, we initialized the model using pre-trained weights obtained from MS-COCO and incorporated transfer learning to fine-tune the model on the Brain MRI dataset for tumor segmentation and classification. The motivation to use pre-trained models is that the model has been trained on massive, publicly accessible datasets, such as ImageNet and MS-COCO, and is therefore capable of learning important features. During training, the initial layers learn low-level features; as the network goes up, the layers can learn task-specific patterns. Thus, when the pre-trained models are trained for a new task such as the segmentation and classification of brain tumors, the training speed and accuracy of the new model increase. As the important image features have already been learned, they do not have to be learned again and are transferred to the new task. This process is known as ‘transfer learning’. For training, the given data are randomly split into training and test sets containing 70% for training and 30% images for testing. The training parameters for the proposed model using custom Mask-RCNN are displayed in Table 1.

### 4.2. Dataset

In this research, we have used two different brain MRI datasets for the evaluation of the proposed technique. The first main dataset used is the ‘Figshare brain tumor dataset’ obtained from [38] and is one of the largest available datasets for brain tumor detection. It contains a total of 3064 real brain MRI samples, collected from 233 different subjects, among which, samples belonging to the meningioma class are 708, pituitary 930, and glioma 1426. The size of each image is 512 × 512 pixels. The second is the Brain MRI Dataset is obtained from [39] and is relatively small. It contains a total of 253 MRI samples of sizes of 845×845 pixels, among which, 155 MRI samples contain tumors. Both datasets are publicly available. The MRI samples are diverse in terms of structural complexity, acquisition angle, devices, noise, and bias field-effect, etc. The reason for using a T1-weighted MRI image dataset is that it is contrast-enhanced. Thus, it provides a better distinction of the areas affected by the tumor, and they are popular for treatment planning.

### 4.3. Evaluation Metrics

**Figure 5:**
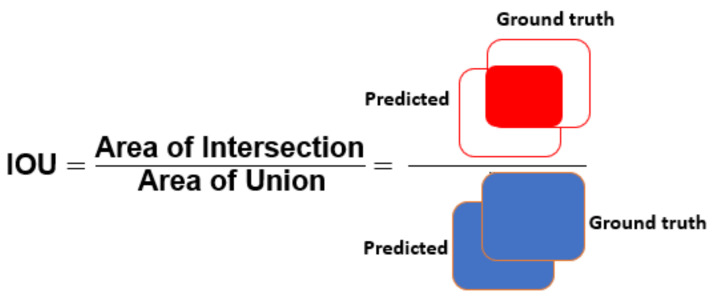
Pictorial representation of IOU.

**Figure 6:**
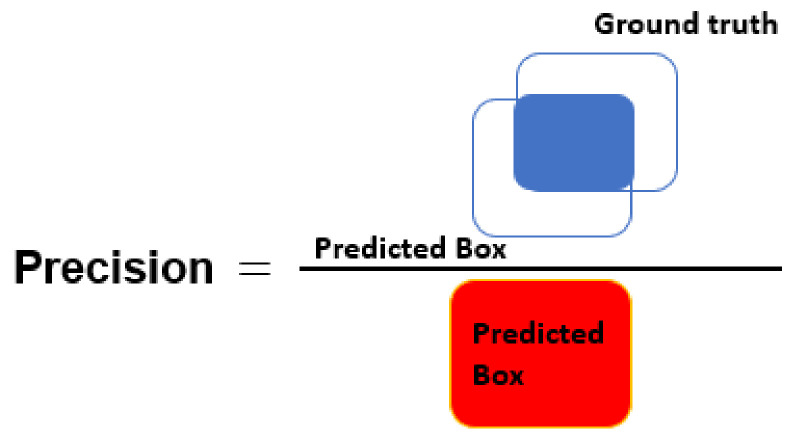
Pictorial representation of precision.

**Figure 7:**
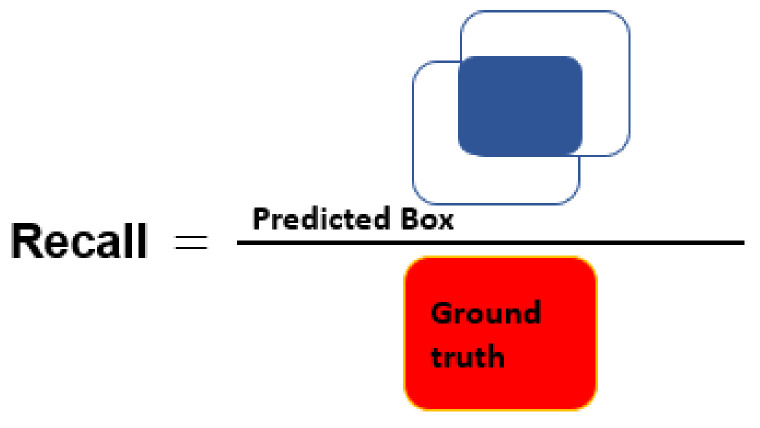
Pictorial representation of recall.

The segmentation results are quantitatively evaluated using parameters such as precision, recall, accuracy (Acc), dice score (DSC), and intersection-over-union (IoU). The equations used for the calculation of Acc, and DSC parameters are given as follows:

(6) $$\mathrm{Acc}=\frac{\left(\mathrm{TP}+\mathrm{TN}\right)}{\left(\mathrm{TP}+\mathrm{TN}+\mathrm{FP}+\mathrm{FN}\right)}$$

(7) $$\mathrm{DSC}=\frac{2\times \mathrm{TP}}{\left(2\times \mathrm{TP}+\mathrm{FN}+\mathrm{FP}\right)}$$

where TP = true positive, TN = true negative, FP = false positive, and FN = false negative cases. Here, note that if the IoU score between the predicted tumor mask and associated GT mask exceeds the threshold value, i.e., 0.7, it will be considered as TP, otherwise it will be considered as FP and it is FN when ground truth mask has no associated predicted tumor mask. Figure 5, Figure 6 and Figure 7 present the pictorial representation of IOU, precision, and recall parameter, respectively.

### 4.4. Experimental Results and Discussion

**Figure 8:**
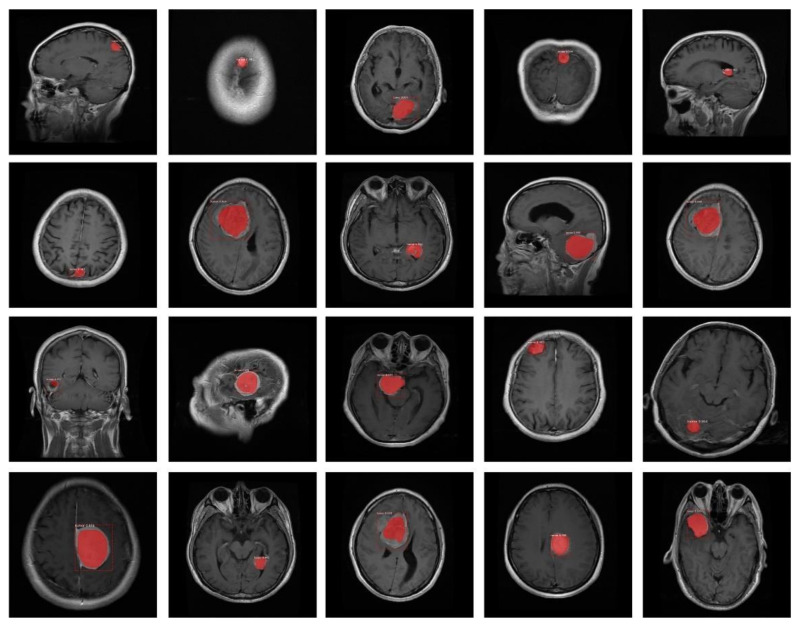
Example segmentation results of high-score-obtaining test images using the proposed method. The red contour shows the predicted tumor mask.

This section contains a detailed analysis and discussion of the obtained results. We have experimented on real MRI images using two datasets [38,39]. The proposed model uses two different deep neural networks, namely ResNet-50 and DenseNet-41 as backbone networks to learn deep features automatically from the training images. However, we obtained better results with DenseNet-41-based Mask-RCNN due to its ability to compute more robust features as compared to ResNet-50. Some of the visual results for custom Mask-RCNN using both datasets are presented in Figure 8. From the figure, we can observe that the presented approach (with DenseNet-41) can more accurately localize the brain tumor from the healthy tissues despite discontinuous or blurry boundaries and artifacts in MRI samples. Moreover, the custom Mask-RCNN (with DenseNet-41) method can precisely segment the brain tumor by overcoming the challenges of location, shape, and size.

**Figure 9:**
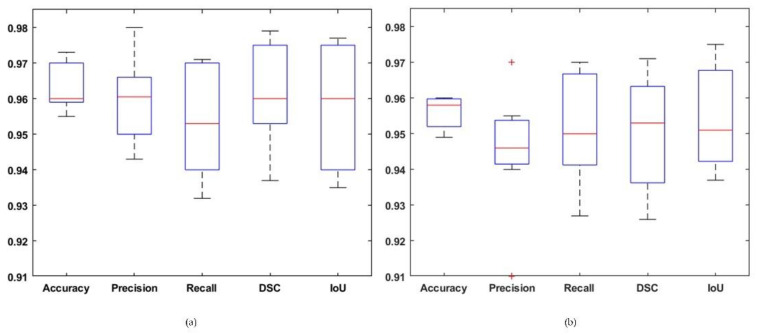
Tumor localization results of the proposed approach over datasets using DenseNet-41 (**a**) Figshare, (**b**) Brain MRI dataset. + sign shows the outer value which is larger than the other values.

**Figure 10:**
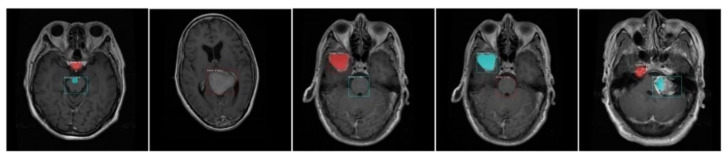
Example of inaccurately localized brain tumor images by the proposed method. The red and blue contour shows the predicted tumor region and respective masks.

As discussed earlier, the obtained results are evaluated using various quantitative measures such as accuracy, precision, recall, DSC, and IoU. To further understand the accuracy of our method, we have drawn a boxplot for evaluation matrices using both datasets, which is shown in Figure 9. The boxplot represents the spread of results into four quartiles, median, and an outlier for all input training images. Part (a) of the figure shows the results obtained over the Figshare dataset while part (b) presents the results for the MRI dataset with the DenseNet-41 based Mask-RCNN framework. From Figure 9, it can be seen that our approach attains better results for the Figshare database as compared to the MRI dataset. The presented approach has achieved an average accuracy and dice score of 95.9% and 0.955 with ResNet-50 and 96.3% and 0.959 using DenseNet-41 over Figshare dataset. The mean average precision (mAP) of the proposed method to localize the brain tumor region at the regression layer is 0.949. In the case of ResNet-50-based Mask-RCNN, the presented technique fails to accurately localize the brain tumor in a few images due to the visual similarity with healthy tissues, as shown in Figure 10. However, for the DenseNet-41-based network, the system has exhibited more accurate results than ResNet-50.

**Figure 11:**
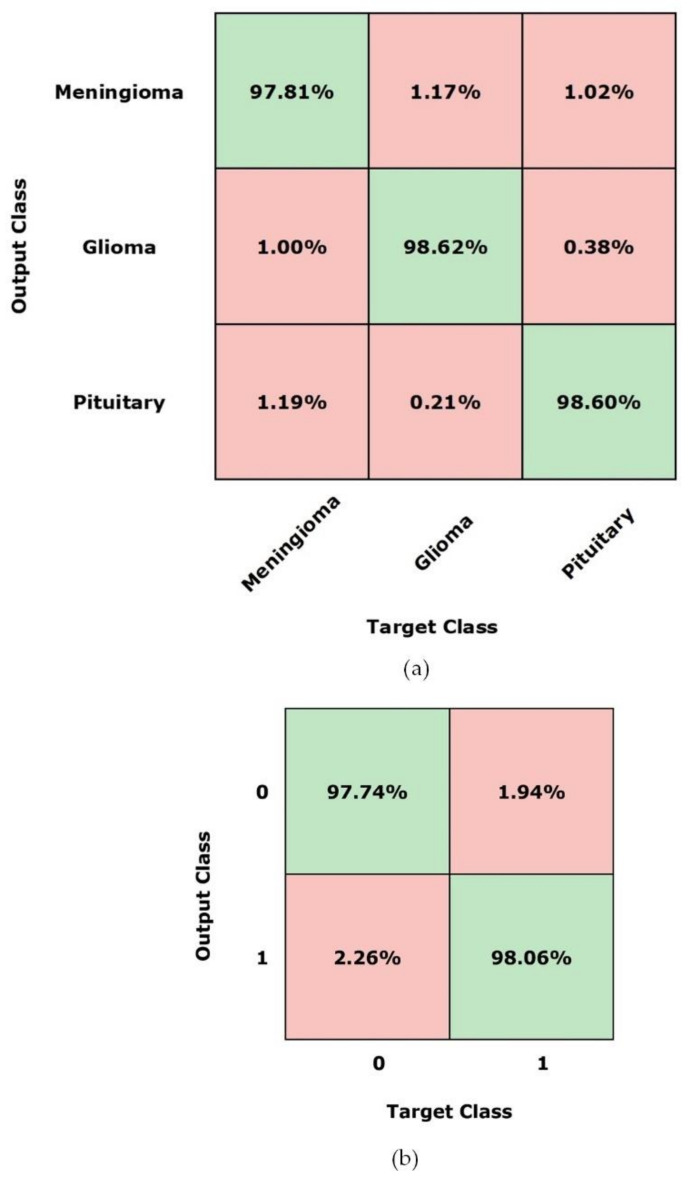
Confusion matrix of the presented technique using DenseNet-41. (**a**) Figshare Brain Tumor Dataset, (**b**) Brain MRI Dataset.

Figure 11 shows the confusion matrices that summarize the classification results of the proposed technique against the ground truth for the DenseNet-41-based network using Figshare [38] and Brain MRI [39] datasets. For both datasets, the presented technique attains better results with DenseNet-41-based Mask-RCNN, as reported in Figure 11. Here, part (a) presents the classification accuracy for the Figshare dataset while part (b) shows the classification results for the MRI dataset. The presented approach obtains an overall classification accuracy of 98.34% for the Figshare dataset while it achieves the accuracy value of 97.90% for the Brain MRI dataset. In the case of the Figshare dataset, the attained classification accuracies for glioma, meningioma, and pituitary tumor types over DenseNet-41 are 98.62%, 97.81%, and 98.60%, respectively, while for the Brain MRI dataset, the presented technique attains the class-wise accuracy of 97.74% and 98.06% for tumor and non-tumor classes, respectively.

#### 4.4.1. Comparison with RCNN-Based Methods

The performance of our technique compared with other region-based segmentation methods, i.e., RCNN [51] and Faster RCNN [56] using Figshare brain tumor dataset [38], is reported in Table 2. The problem with RCNN methods is that they require significantly too much time to train the model as these techniques randomly generate around 2000 region proposals per image for classification. Additionally, there is no learning process at the region proposal generation step because a fixed selective search algorithm is used, which leads to the generation of false candidate region proposals. Furthermore, the processing time for the test image is approximately 47 s, which is inadequate for obtaining results in real-time. Faster RCNN extracts the region proposals automatically by introducing the region proposal network and shares the CL among class and bounding box network to expedite the process and reduce the computation cost. Faster RCNN and Mask-RCNN give results in real-time, i.e., approximately 0.23 and 0.2 s, respectively, inference time per test image. The advantage of using Mask-RCNN over Faster RCNN is the automated segmentation of the brain tumor along with localization, i.e., it defines the tumor location and draws a high-quality segmentation mask of the tumor region along with classification. Mask-RCNN delineates RoIs automatically and extracts the features from the images layer by layer without providing the features previously. This gives an advantage of detecting the tumor comprehensively besides analyzing the single features. Moreover, it is easier to train and requires a negligible additional overhead as compared to Faster RCNN. Moreover, the presented work with DenseNet-41 is more robust as compared to ResNet-50 due to its dense connections, which result in calculating a more accurate set of image features. Furthermore, the DenseNet-41-based Mask-RCNN is computationally more efficient due to its small number of parameters.

#### 4.4.2. Comparison with Other Segmentation Techniques

In this section, we compare our proposed model with the other segmentation techniques using the Figshare brain tumor dataset [38] which is one of the largest online available datasets. For performance evaluation, we compared the average highest results of our presented technique with the average results reported in these studies [61,62,63,64,65]. For the presented technique, we have shown the results for the DenseNet-41-based Mask-RCNN framework as we obtained better performance on it as compared to the ResNet-50 framework.

Table 3 shows a quantitative comparison using different performance measurement metrics such as mean IoU, dice score, and accuracy. Sheela et al. [61] proposed a pixel-based radius contraction and expansion (RCE) technique that uses an active contour model and fuzzy c-means for tumor segmentation. However, the performance depends on the threshold value set for extracting the region of interest. They obtained an average accuracy of 0.91. In [62], the authors proposed a cascaded dual scale LinkNet (CDSL Net), an end-to-end encoder–decoder-based architecture that uses multi-scale inputs for feature concatenation with corresponding layers in the network to perform brain tumor segmentation. They obtained a dice score of 0.8003. Gunasekara et al. [63] employed a faster RCNN model for tumor classification and then a Chan–Vese active contour algorithm for segmentation. They achieved an average dice score of 0.92 and an accuracy of 92.31 for tumor segmentation and classification, respectively. However, the authors considered only the axial MR images of glioma and meningioma brain tumors. In [64], the authors proposed a multi-scale CNN model that processes the input MR image in three different spatial scales using multiple processing pathways. They achieved an average accuracy of 0.973 and a dice score of 0.828 for classification and segmentation, respectively. In [65], we proposed a Mask-RCNN model with ResNet101 as a backbone for brain tumor detection and segmentation only. Our method in [65] achieved a dice score of 0.950; however, this method is computationally expensive due to the large number of parameters.

As can be seen in Table 3, the proposed work does better than the other state-of-the-art techniques by achieving a dice sore of 0.959, a mean IoU of 0.957, and an overall average accuracy of 96.3% using DenseNet-41 for three different kinds of tumors. The proposed technique uses deep features that are more discriminating, reliable, and provide a more effective representation of tumor regions over other methods such as [61], which employs the hand-crafted features and is unable to better represent the tumor region due to structural complexities. Moreover, in some existing methods [62,64] segmentation is applied directly to the entire image, which results in misclassification due to the complex background (i.e., brain tissues overlapping with tumor boundary, MRI artifacts, etc.), which thus reduces the accuracy of the segmentation. The method in [63] employs a region-based method for tumor localization and requires further processing for tumor segmentation. Unlike these methods, our model performs segmentation on the localized RoIs, which limits the space of segmentation and uses the RoIAlign layer, which ultimately improves the accuracy of the segmentation result. Moreover, our method achieves comparable performance with [65] while using less computational resources.

#### 4.4.3. Comparison with Other Classification Techniques

In this section, we present the comparison of classification results of our approach with results obtained by previous works over the same dataset [38]. Table 4 shows the comparison of tumor classification results with existing approaches in terms of average accuracy. In Table 4, we have presented the highest results of our proposed framework obtained using the DenseNet-41 backbone. In [66], the authors employed a pre-trained GoogleNet model by using transfer learning for feature extraction. The obtained features are classified by using three different classifiers: Softmax, SVM, and KNN, and achieved an accuracy of 97.1%. Swati et al. [67] utilized various DL models: AlexNet, VGG16, and VGG19. They fine-tuned VGG16 and VGG19 in a block-wise manner and AlexNet using the traditional layer-wise approach. The results showed VGG19 achieved better performance, with an average accuracy of 94.82 for brain tumor classification. Huang et al. [68] proposed a deep CNN model with a modified activation function for brain tumor classification. The CNN model is constructed automatically by a network generator based on three different graph generation algorithms. The activation function is composed of Gaussian error linear units (GeLUs) and rectified linear units (ReLUs). The method achieved an accuracy of 95.49. However, the proposed approach is computationally complex due to network size. In [69], the authors proposed a hybrid feature extraction approach by using a PCA-based normalized GIST descriptor with a regularized extreme learning machine classifier. They obtained an overall accuracy of 94.93%. However, these approaches [66,69] require preprocessed input images, i.e., the normalized pixel values from 0 to 1. In [70], a DL model named BrainMRNet employed three different processing methods such as attention modules, the hypercolumn technique, and residual blocks for brain tumor classification. Besides, the segmentation of the brain using the Otsu method is also performed to determine the lobe region (i.e., left, or right) with more concentrated tumorous cells. They obtained an accuracy of 97.69% for classification.

From Table 4, it can be seen that in comparison to existing techniques, the proposed approach shows an improved overall accuracy of 98.34% for brain tumor type classification. The above-mentioned approaches [66,67,68] extract features from the whole image which may result in the misclassification of tumor type due to the complex nature of the tumor, i.e., overlapping boundaries and MRI artifacts. In [69], hand-crafted features are employed that are less discriminative and robust. The technique in [70] achieves results comparable to our approach; however, due to the hypercolumn technique, the model leads to overfitting and is computationally expensive, whereas the proposed approach employs deep features that are more discriminative and reliable. Moreover, region-based CNN first localizes the tumor region (RoI) and performs classification that results in improved accuracy.

## 5. Conclusions

In this work, we introduced a DL technique, namely Mask-RCNN with two backbones, ResNet-50 and DenseNet-41, for precise and automated segmentation of brain tumor regions from MRI images. We obtained better segmentation and classification results for DenseNet-41 based Mask-RCNN as compared to the ResNet-50 network, due to its dense connections which result in more robust image feature calculations. Comparative experimental results show that our proposed method more precisely delineates the tumor region and can serve as a new automated tool for diagnostic purposes. Moreover, as compared to state-of-the-art models, our Custom Mask-RCNN can compute deep features with effective representations of brain tumors. In future, we aim to perform classification along with segmentation of brain tumors using more challenging datasets. We also plan to evaluate the robustness of our Custom Mask-RCNN for other medical image analyses applications such as eye disease detection, finger skin recognition, skin cancer, and COVID detection. Furthermore, we aim to increase training samples and optimize hyper-parameters to further improve the accuracy of the model.

## Figures and Tables

**Table 1:** Training parameters of the presented technique.

Parameters	Value
Epochs	45
Learning rate	0.001
IoU Threshold	0.70

**Table 2:** Performance comparison of our technique with other RCNN approaches.

Method	Evaluation Metrics				
	Accuracy	mAP	Dice	Sensitivity	Time(s)
RCNN [51]	0.920	0.910	0.870	0.950	0.47
Faster RCNN [56]	0.940	0.940	0.910	0.940	0.25
Proposed (Resnet-50)	0.959	0.946	0.955	0.953	0.20
Proposed (Densenet-41)	0.963	0.949	0.959	0.953	0.20

**Table 3:** Comparison of the presented method with other segmentation techniques.

Technique	Segmentation Method	Evaluation Metrics		
		Mean IoU	Dice	Accuracy
Sobhaninia et al. [62]	Cascaded CNN	0.907	0.800	-
Gunasekara et al. [63]	Faster RCNN and ChanVese active contour	-	0.920	94.6
Sheela et al. [61]	Active Contour and Fuzzy-C-Means	-	0.665	91.0
Díaz-Pernas et al. [64]	Multi-scale CNN	-	0.828	-
Masood et al. [65]	Traditional Mask-RCNN	0.950	0.950	95.1
Proposed method	Mask-RCNN (ResNet-50)	0.951	0.955	95.9
	Mask-RCNN(DenseNet-41)	0.957	0.959	96.3

**Table 4:** Comparison of our method with other classification techniques.

Technique	Classification Method	Acc (%)
Deepak et al. [66]	GoogLeNet and SVM	97.10
Swati et al. [67]	VGG19	94.82
Huang et al. [68]	CCN based on complex networks	95.49
Gumaei et al. [69]	GIST descriptor and ELM	94.93
BrainMRNet [70]	Attention module, Hypercolumn technique, and Residual block	97.69
Proposed method	Custom Mask-RCNN	98.34

## Data Availability

Data sharing not applicable to this article as authors have used publicly available datasets, whose details are included in the “experimental results and discussions” section of this article. Please contact the authors for further requests.
